# Inhibitory Effects of Glycyrrhetinic Acid on the Delayed Rectifier Potassium Current in Guinea Pig Ventricular Myocytes and HERG Channel

**DOI:** 10.1155/2013/481830

**Published:** 2013-08-29

**Authors:** Delin Wu, Linqing Jiang, Hongjin Wu, Shengqi Wang, Sidao Zheng, Jiyuan Yang, Yuna Liu, Jianxun Ren, Xianbing Chen

**Affiliations:** ^1^Beijing Hospital of Integrated Traditional Chinese and Western Medicine, Beijing 100039, China; ^2^Beijing Institute of Radiation Medicine, Beijing 100850, China

## Abstract

*Background*. Licorice has long been used to treat many ailments including cardiovascular disorders in China. Recent studies have shown that the cardiac actions of licorice can be attributed to its active component, glycyrrhetinic acid (GA). However, the mechanism of action remains poorly understood. *Aim*. The effects of GA on the delayed rectifier potassium current (*I*_K_), the rapidly activating (*I*_Kr_) and slowly activating (*I*_Ks_) components of *I*_K_, and the HERG K^+^ channel expressed in HEK-293 cells were investigated. *Materials and Methods*. Single ventricular myocytes were isolated from guinea pig myocardium using enzymolysis. The wild type HERG gene was stably expressed in HEK293 cells. Whole-cell patch clamping was used to record *I*_K_ (*I*_Kr_, *I*_Ks_) and the HERG K^+^ current. *Results*. GA (1, 5, and 10 **μ**M) inhibited *I*_K_ (*I*_Kr_, *I*_Ks_) and the HERG K^+^ current in a concentration-dependent manner. *Conclusion*. GA significantly inhibited the potassium currents in a dose- and voltage-dependent manner, suggesting that it exerts its antiarrhythmic action through the prolongation of APD and ERP owing to the inhibition of *I*_K_ (*I*_Kr_, *I*_Ks_) and HERG K^+^ channel.

## 1. Introduction

Cardiac arrhythmias are associated with significant morbidity and mortality in developed and developing countries. Although nonpharmacologic approaches, ablative therapy, and implantable defibrillators, for example, are being used more and more commonly as complementary and alternative interventions for the treatment of cardiac arrhythmias, drug therapy was traditionally the mainstay of arrhythmia treatment. Improved understanding of the cardiomyocyte ion channels holds the promise of identifying novel targets for the treatment of cardiac arrhythmias. Furthermore, the limited efficacy and the potency of provoking life-threatening arrhythmias of present drugs have generated interest in finding new antiarrhythmic agents [1]. 

Potassium channels constitute the most abundant family of ion channels involved in cardiac physiological or pathophysiological processes, disruptions of which could give rise to prolongation of the action potential and potentiate occurrence of lethal arrhythmias subsequently [2, 3]. In the hearts of many mammalian species including humans, delayed rectifier K^+^ current (*I*_K_), the major outward potassium current responsible for ventricular repolarization, can be divided into at least two different components, rapidly activating (*I*_Kr_) and slowly activating (*I*_Ks_). The human ether-à-go-go-related gene (HERG) encodes the *α*-subunit for the rapid delayed rectifier potassium channel (*I*_Kr_) in cardiac myocytes. *I*_Kr_ and *I*_Ks_ are pivotal in cardiac repolarization, especially in the later phases of the action potential [4, 5]. Although the structural and functional aspects of potassium channels have been widely examined, only a few K^+^ channel modulators are being used clinically at present for the unwanted adverse effects [2, 6]. As an alternative to find new antiarrhythmic agents, there has been increased interest in investing natural compounds that are effective against cardiac arrhythmia.

*Glycyrrhiza* radix is a commonly prescribed herb to prevent palpitations in Chinese traditional medicine for about 2000 years, derived from the dried roots and rhizomes of *Glycyrrhiza uralensis, G. glabra*, and *G. inflata*. Glycyrrhizin, the major constituent of *G. glabra*, is a glycoside, which occurs as an admixture of sodium, potassium and calcium salts [7]. Orally administered, glycyrrhizin is poorly absorbed by the intestinal tract and is hydrolyzed by *β*-D-glucuronidase-containing intestinal bacteria to yield two molecules of D-glucuronic acid and the aglycone glycyrrhetinic acid (GA), a pentacyclic triterpene [8]. If intravenously administered, GA is metabolized in the liver by lysosomal *β*-D-glucuronidase to the 3-monoglucuronide of glycyrrhetinic acid. This metabolite is excreted via the bile into the intestine, where it is transformed by bacteria into GA, which can be reabsorbed, causing a pronounced delay in terminal plasma clearance [9].

The use of *Glycyrrhiza uralensis* as a pharmacological remedy dates back far into the past [10]. Various pharmacological properties of licorice have been proved including cardioprotective [11, 12], antiulcer, anti-inflammatory, spasmolytic, antioxidative, contravariant, antiviral, anticancer, and hepatoprotective effects, as well as eliminating phlegm and reinforcing memory [7, 13–15]. Many components have been isolated from licorice including triterpene saponins, flavonoids, isoflavonoids, and chalcones. Triterpene saponins are the main components of *Glycyrrhiza* radix and its pharmacological activities are comparatively well understood and clear.

Recently, wide-ranging studies have provided evidence that GA is cardioprotective; this action involves different pathways. In rat cardiac mitochondria [16], GA was shown to increase permeability and concomitant release of proapoptotic factors. In particular, GA, acting as a gap junction inhibitor, influences connexin 43, the major gap junction-forming protein in adult cardiac ventricles and a regulator of mitochondrial function [17]. Studies have demonstrated that GA and its derivatives affect the inotropic, lusitropic, chronotropic, and coronary performances of the mammalian heart and the signal transduction pathways that could be involved [9]. Furthermore, research has demonstrated that GA reduces cardiac sodium currents [18], particularly the late *I*_Na_. These findings might help to elucidate the traditional use of licorice in therapy for cardiovascular disorders [19]. It has also been reported that 18*β*-GA has significant potential for development as a novel antiarrhythmic agent and for treating myocardial ischemia by preferentially blocking the *I*_Na,L_ [20]. However, the effects of GA on the potassium channel, especially the rapid and slow components of the delayed rectifier potassium current, are not clearly defined.

In this study, we investigated the effects of GA on the *I*_K_ (*I*_Kr_, *I*_Ks_) in guinea pig ventricular myocytes. We also extended our study to investigate the effects of GA on the human ether-à-go-go-related gene (HERG) K^+^ channel current expressed in HEK-293 cells. Our study could provide theoretical support for developing the significant potential of GA as a novel antiarrhythmic agent.

## 2. Materials and Methods

### 2.1. Materials

Glycyrrhetinic acid ((GA) molecular weight 470.6, purity > 99%) was obtained from the Chinese Biological Product Assay Institute (Beijing, China). The white powder was dissolved in dimethylsulfoxide (DMSO) as stock. The percentage of DMSO in the final solution was less than 0.1%. Collagenase II was obtained from Worthington Biochemical Corporation (Lakewood, NJ, USA). Protease XIV, Na_2_ATP, MgATP, CdCl_2_, L-Glutamic acid, Taurine, and EGTA were purchased from Sigma Co. Other reagents were of analytical reagent grade. Male guinea pigs weighing 300–350 g were provided by the Vital river Company, Certificate no.: SCXK (Jing) 2006-0009. 

### 2.2. Solutions

Ca^2+^-free Tyrode's solution (mM) contained NaCl 137, KCl 5.4, NaH_2_PO4 1.2, MgCl_2_ 1.2, HEPES 10, glucose 10, and Taurine 10 (adjusted to pH 7.36–7.38 with NaOH).

Kraft-Brühe (KB) solution (mM) contained L-glutamic acid 50, KCl 40, KH_2_PO4 20, Taurine 20, MgCl_2_ 3.0, KOH 70, EGTA 0.5, HEPES 10, and glucose 10 (adjusted to pH 7.25 with KOH).

For *I*_K_ (*I*_Kr_, *I*_Ks_) recordings from ventricular myocytes, the control bath solution contained (mM) NaCl 135, KCl 5.4, CaCl_2_ 1.0, CdCl_2_ 0.2, NaH_2_PO4 0.33, MgCl_2_ 1.0, HEPES 5, and glucose 5 (adjusted to pH 7.36–7.38 with NaOH). Drugs were added to the bath solution. Pipettes had tip resistances of 1.5–2 MΩ when filled with a solution containing (mM) KCl 140, MgCl_2_ 1, HEPES 5, EGTA 10, and Na_2_ATP 2 (adjusted to pH 7.25 with KOH).

To record HERG activity in the HEK 293 cells, the internal solution contained (mM) KCl 130, MgCl_2_ 1, HEPES 10, EGTA 10, and MgATP 5 (adjusted to pH 7.25 with KOH). The external solution contained (mM) NaCl 136, KCl 5.4, CaCl_2_ 1, MgCl_2_ 1, HEPES 10, and glucose 10 (adjusted to pH 7.36–7.38 with NaOH).

### 2.3. Isolation of Single Ventricular Myocytes

Adult guinea pigs were fully anesthetized by intraperitoneal injection of urethane (40 mg/kg). The hearts were quickly removed and mounted on a Langendorff column, and cardiac myocytes were isolated as follows. The heart was dissected and rinsed in cold oxygenated Ca^2+^-free Tyrode's solution and then perfused in the Langendorff apparatus at 37°C. Perfusion with Ca^2+^-free Tyrode's solution for 5 min was followed first by 25 ± 5 min perfusion with low Ca^2+^ (0.1 mmol/L) Tyrode's solution containing 0.04–0.06 g/L collagenase and 0.5–0.8 g/L BSA and then 5 min perfusion with collagenase-free Tyrode's solution containing 0.5–0.8 g/L BSA. The heart was then minced and the cells were filtered through 200 *μ*m nylon mesh, resuspended in Kraft-Brühe (KB) solution, and stored at room temperature (22–25°C) until use.

### 2.4. Cell Culture

The HEK293 cells that stably expressed the wild type HERG gene were kindly provided by Professor Xiaoyan Liu (Academy of Military Medical Sciences, Beijing, China). Dulbecco's Modified Eagle's Medium (DMEM, Hyclone) supplemented with 10% (v/v) fetal bovine serum (FBS, Gibco) and 1 mg/mL geneticin (G-418, Gibco), the cultures were passed every 3–5 days by use of a brief trypsin treatment. The cells were maintained at 37°C in 5% CO_2_ and plated on a glass culture dish 2-3 days before electrophysiological experiments. 

### 2.5. Electrophysiological Recordings

All currents were recorded using the conventional whole-cell patch-clamp technique. Borosilicate glass electrodes had tip resistances of 1–3 MΩ when filled with the pipette solution. All experiments were performed at room temperature (22-23°C) using an Axopatch 200B amplifier (Axon Instrument, USA). Adequate series resistances (less than five times the pipette resistances) were usually attained within 10 min after the gigaohm seal was formed. Measurements were taken using an Axopatch 200B amplifier (Axon Instruments). The current signals were filtered via a 4 kHz, 4-pole low-pass filter and digitized with an AD-DA converter (Digidata 1440, Axon Instruments) for subsequent analysis using pCLAMP 10.0 software.

### 2.6. Statistical Analysis

The data were analyzed with the use of Data Processing System (Version 7.05). All data are expressed as means ± SEM. Paired Student's *t*-tests were used for statistical comparisons when appropriate, and differences were considered significant at *P* < 0.05.

## 3. Results

### 3.1. Effect of GA on the *I*_*K*_ of Guinea Pig Ventricular Myocytes

**Figure 1:**
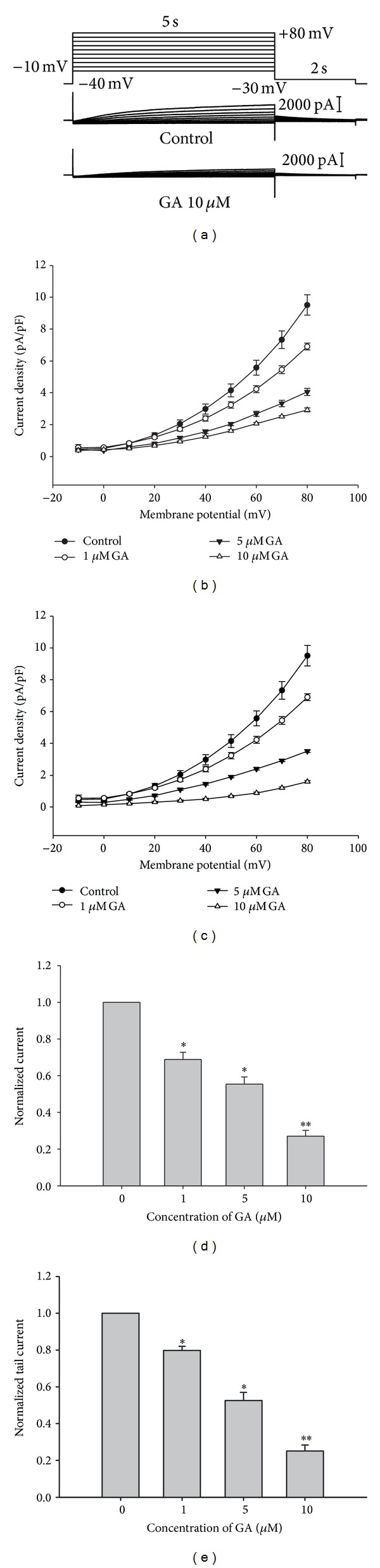
Effect of GA on the *I*_K_ of guinea pig ventricular myocytes. (a) Current traces elicited by 5 s test pulses from −10 to +80 mV with 10 mV increments, and tail currents recorded at −30 mV for 2 s. Representative traces of *I*_K_ recorded from the same cell under control condition and after applying GA. Current-voltage relationship of *I*_K,step_ (b) and *I*_K,tail_ (c) observed in five cells tested in the absence and presence of GA (1, 5, and 10 *μ*M). Summary of the effects of GA (1, 5, and 10 *μ*M) on *I*_K,step_ (d) and *I*_K,tail_ (e), normalized relative to the control current (*n* = 5, *P < 0.05, **P < 0.01).

We first tested the effect of GA on *I*_K_ using guinea pig ventricular myocytes. *I*_K_ were recorded by applying voltage pulses ranging from −10 to +80 mV for 5 s from the holding potential of −40 mV, and the repolarization potential was maintained at a constant −30 mV for the *I*_K,tail_ analysis.  Figure 1(a) shows an example of a voltage-clamp recording from a single ventricular myocyte, with representative current traces given under control conditions and after exposure to 10 *μ*M GA. Under control conditions, the depolarizing steps activated time-dependent outward currents. The amplitude of the outward currents measured at the end of the pulse (*I*_K_) increased with increasingly positive voltage steps. The current-voltage relationships for *I*_K,step_ and *I*_K,tail_ obtained at various concentrations of GA are shown in Figures 1(b) and 1(c). As the concentration of GA increased, the amplitude of *I*_K,step_ and *I*_K,tail_ decreased dose dependently (Figures 1(d) and 1(e)). The *I*_K,step_ measured at +80 mV was 6.97 ± 0.34 pA/pF under control conditions and decreased to 4.93 ± 0.51 pA/pF, 3.35 ± 0.55 pA/pF, and 2.12 ± 0.29 pA/pF after application of 1, 5, and 10 *μ*M GA, respectively (*n* = 5, *P* < 0.05). Meanwhile, the *I*_K,tail_ evoked after repolarization to −30 mV was 1.57 ± 0.11 pA/pF under control conditions and decreased to 1.24 ± 0.12 pA/pF, 0.67 ± 0.15 pA/pF and 0.48 ± 0.06 pA/pF in the presence of 1, 5 and 10 *μ*M GA, respectively (*n* = 5, *P* < 0.05). These results show that GA dose-dependently blocked *I*_K,step_ and *I*_K,tail_.

### 3.2. Effect of GA on *I*_Kr_ and *I*_Ks_ in Guinea Pig Ventricular Myocytes

**Figure 2:**
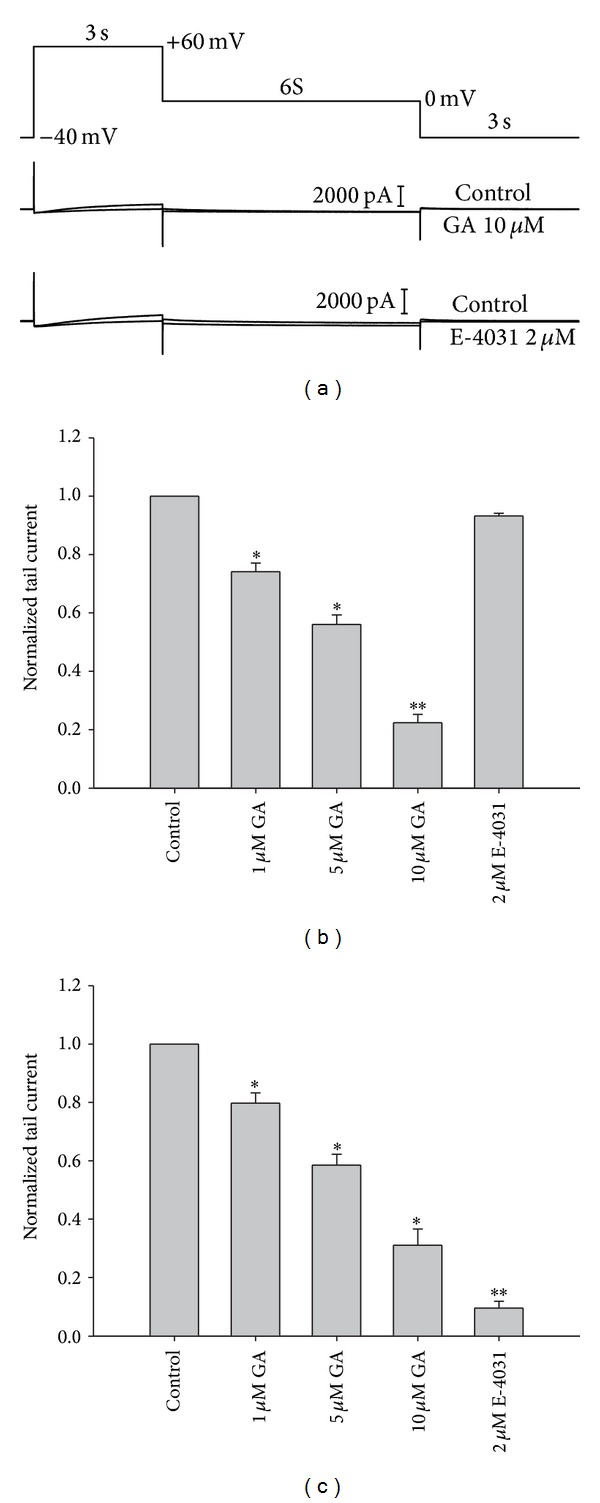
Effect of GA on the slow and rapid components of the delayed rectifier K^+^ current in guinea pig ventricular myocytes. (a) Representative traces of the rapid (*I*_Kr_) and slow (*I*_Ks_) components of the delayed rectifier K^+^ channel tail currents before and after treatment with 10 *μ*M GA and 2 *μ*M E-4031. The effects of 1, 5, and 10 *μ*M GA and 2 *μ*M E-4031 on *I*_Ks,tail_ (b) and *I*_Kr,tail_ (c), normalized relative to the control current (*n* = 5, *P* < 0.05). The tail current amplitudes were differences between peak outward current and the steady state current at the end of the repolarizing voltage pulses. (*P < 0.05, **P < 0.01).

To investigate the effects of GA on the rapid and slow components of the delayed rectifier currents in guinea-pig ventricular myocytes, we used a voltage clamp protocol designed to separate the currents electrophysiologically. The holding potential was maintained at −40 mV. Our results revealed that depolarization to +60 mV activated both *I*_Kr_ and *I*_Ks_. Repolarization to 0 mV revealed *I*_Ks_ as a deactivating *I*_Ks,tail_, and subsequent repolarization to −40 mV resulted in deactivation of *I*_Kr_. We confirmed that 2 *μ*M E-4031, a selective blocker of *I*_Kr_, blocked the rapid component of the delayed rectifier K^+^ current but had no effect on *I*_Ks_ (Figure 2). As shown in Figures 2(b) and 2(c), 1, 5, and 10 *μ*M GA dose dependently inhibited *I*_Ks,tail_ and *I*_Kr,tail_. In the cells examined, *I*_Ks,tail_ measured at 0 mV was 0.59 ± 0.03 pA/pF under control conditions and decreased to 0.53 ± 0.06 pA/pF, 0.33 ± 0.06 pA/pF, and 0.16 ± 0.03 pA/pF after application of 1, 5, and 10 *μ*M GA, respectively (*n* = 5, *P* < 0.05). The *I*_Kr,tail_ evoked after repolarization to −40 mV was 1.57 ± 0.11 pA/pF under control conditions and decreased to 0.57 ± 0.06 pA/pF, 0.29 ± 0.02 pA/pF and 0.21 ± 0.05 pA/pF in the presence of 1, 5 and 10 *μ*M GA, respectively (*n* = 5, *P* < 0.05). This result shows that GA blocks the rapid and slow components of delayed rectifier K^+^ current.

### 3.3. Inhibition of HERG K^+^ Currents and Concentration-Dependent Block by GA

**Figure 3:**
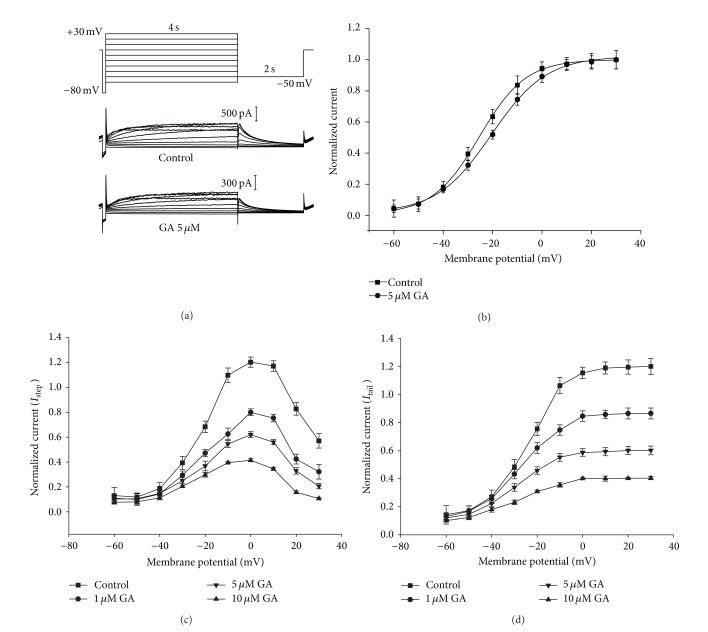
Current-voltage relationship for HERG channels and blockade by GA. (a) HERG currents are illustrated under control conditions and in the presence of 5 *μ*M GA recorded using the pulse protocol. (b) Voltage-dependent activation curves for the control and following GA exposure. Tail currents under control conditions and in the presence of 5 *μ*M GA were normalized and fitted to the Boltzmann sigmoidal function. There were no significant differences in half-activation voltage (*V*_1/2_) of 5 *μ*M compared with the control (*P* > 0.05, *n* = 6). Data were expressed as mean ± SEM. Statistical comparisons were made using a two-tailed Student's *t*-test. Normalized (to respective control values) *I*-*V* relationships for current measured at the end of depolarizing steps (c) and tail currents (d) in the control and the presence of 1 *μ*M, 5 *μ*M, and 10 *μ*M GA. Data are expressed as mean ± SEM; *n* = 6. Statistical comparisons were made using a two-tailed Student's *t*-test (*P* < 0.05, *n* = 6).

The HERG current was elicited from the holding potential of −80 mV by test pulses ranging from −60 to +30 mV in 10 mV steps. Each test pulse was followed by a repolarization step to −50 mV, which evoked large, slowly decaying outward tail currents. Currents were recorded first under control conditions, and then GA (5 *μ*M) was washed into the bath for 10 min, with the cell kept at the holding potential, before current recording commenced in the presence of the drug (Figure 3(a)). In the absence of drug, the *I*-*V* relationship exhibited the characteristic bell-shaped curve increasing from −40 to 0 mV, and owing to the fast C-type inactivation of HERG channels, it decreased with further depolarization. GA reduced both the HERG current (*I*_step_) during the test potentials and the tail current (*I*_tail_) after a test pulse to 0 mV (Figures 3(c) and 3(d)). GA inhibited the tail current at all potentials, although voltage dependence was evident with a significantly weaker block at more negative potentials (Figure 3(d)).

**Figure 4:**
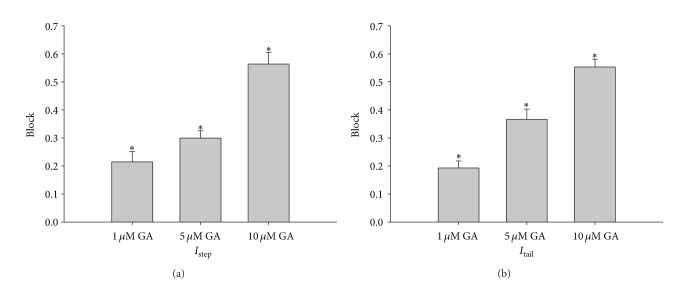
Inhibitory effect of GA on HERG current. Mean fractional block of HERG at a test potential of 0 mV at GA concentrations of 1, 5, and 10 *μ*M was determined in HEK293 cells that stably expressed HERG channels.

GA blocked the HERG currents and the tail current in a concentration-dependent manner. The stimulus protocol used is illustrated in Figure 3(a). At GA concentrations of 1, 5, and 10 *μ*M, the HERG current amplitude at 0 mV and the peak tail current amplitude were measured, normalized for each cell to the control value and then averaged (*n* = 5). The fractional block of *I*_step_ was 20.46 ± 2.7% in the 1 mM group (*P* < 0.05), 29.93 ± 3.1% in the 5 mM group (*P* < 0.05), and 56.38 ± 4.2% in the 10 mM group (*P* < 0.05); the corresponding fractional blocks of *I*_tail_ were 19.26 ± 2.5% (*P* < 0.05), 36.57 ± 3.0% (*P* < 0.05), and 55.29 ± 3.6% (*P* < 0.05), respectively (Figure 4).

### 3.4. Effects of GA on HERG Channel Kinetics

Drugs that block ion channels often alter the voltage dependence or kinetics of channel gating. Therefore, we examined the effects of GA on the voltage dependence of activation and rectification and on the kinetics of inactivation and deactivation. The activation curves were constructed by normalizing the tail currents recorded with the protocol used in Figure 3(a). The activation curve showed that the threshold voltage for HERG current activation was close to −50 mV and that it was fully activated with voltage steps to −10 mV. The rate of activation was similar before and after exposure to 5 *μ*M GA. *V*_1/2_ values were −19.83 ± 2.36 mV in the control and −22.56 ± 1.84 mV in GA (*P* > 0.05, *n* = 5). Thus, 5 *μ*M GA had little effect on the voltage dependence of activation (Figure 3(b)).

**Figure 5:**
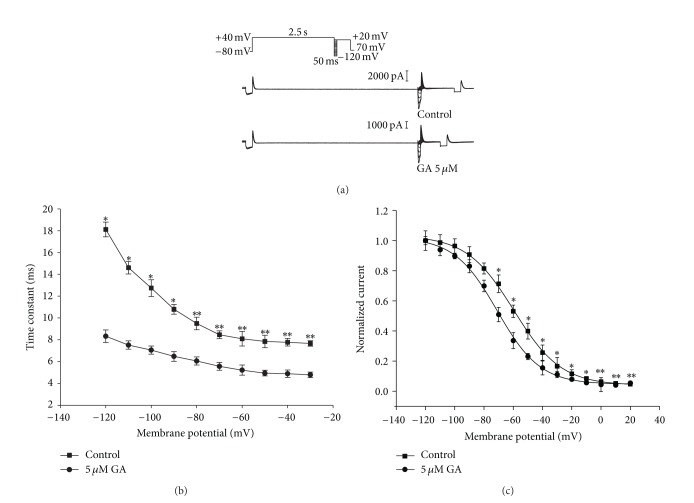
Effect of GA on normalized steady-state inactivation curves. (a) Representative active current traces for steady-state inactivation using a three-pulse protocol with various interpulse repolarization levels (from −120 mV to 20 mV). (b) The time constant of inactivation was significantly reduced at all membrane potential values (*P* < 0.05, *n* = 5). (c) Normalized steady-state inactivation curves before and after 5 *μ*M GA application (*P* < 0.05, *n* = 5). Data were expressed as mean ± SEM. Solid lines represent fits to the Boltzmann sigmoidal function. Statistical comparisons were made using a two-tailed Student's *t*-test. (*P < 0.05,**P < 0.01).

To measure inactivation, a special protocol was used that inactivated the channel at a holding potential of 40 mV, recovered the channel from inactivation at various potentials from −120 to 20 mV in 10 mV steps, and measured the resulting peak outward current at constant 20 mV as a measure of steady-state inactivation (Figure 5(a)). Mean data from five cells were fitted to a Boltzmann function, yielding inactivation *V*_1/2_ values of −51.27 ± 3.21 mV in the control and −62.68 ± 2.85 mV in 5 *μ*M GA (*P* < 0.05, *n* = 5) (Figure 5(c)). The time course of the development of inactivation was also assessed. Monoexponential curve fitting of the inactivation time-course yielded time constant values (Figure 5(b); *n* = 5). From −120 to −20 mV, the time constants of inactivation were significantly lower in the presence of 5 *μ*M GA (*P* < 0.05, *n* = 5) than in the absence of the drug.

**Figure 6:**
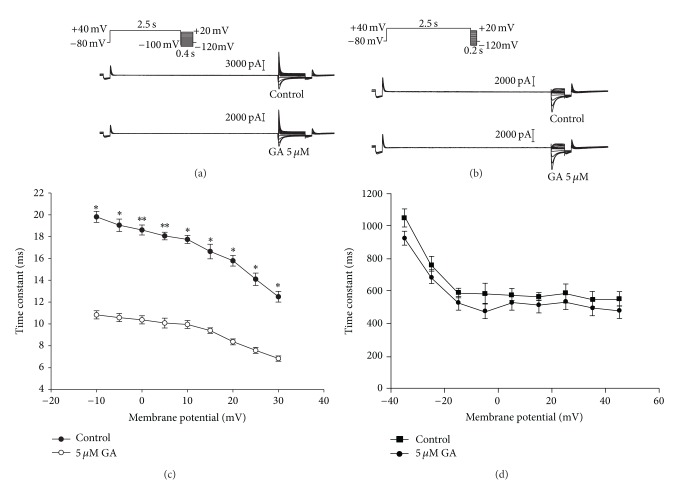
Effect of GA on kinetic time constants. (a) Representative current traces for the onset of inactivation of the HERG current using the protocol are shown on the figure. The time constants for the onset of inactivation were obtained by fitting the exponential function to the decaying current traces during the third pulse of the protocol. (b) Representative current traces for HERG current recovery from inactivation elicited by the protocol are shown on the figure. (c) Time constants for onset of inactivation were plotted against membrane potential. Data were expressed as mean ± SEM (*P* < 0.05, *n* = 5). (d) Time constants for deactivation in the absence or presence of 5 *μ*M GA (*P* > 0.05, *n* = 5). Solid lines represent fits of a single exponential function to the descending phase of the tail current. Data were expressed as mean ± SEM, and statistical comparisons were made using a two-tailed Student's *t*-test. (*P < 0.05, **P < 0.01).

The effect of GA on the onset of inactivation of the HERG current was investigated using a three-pulse protocol. The channels were first inactivated by clamping the membrane at 40 mV followed by a prepulse to −100 mV. This prepulse was sufficiently long to allow rapid recovery of channels from inactivation but short enough to prevent significant channel deactivation. Following recovery from the prepulse, a series of test pulses were delivered to potentials ranging from −120 to 20 mV, resulting in outward inactivating currents (Figure 6(a)). The time constants for the onset of inactivation were obtained by fitting exponential functions to the decaying current traces during the third pulse of the protocol and were significantly decreased following perfusion with 5 *μ*M GA (*P* < 0.05, *n* = 5) (Figure 6(c)). 

To determine recovery from inactivation, the fully activated *I*-*V* protocol shown in Figure 6(b) was used: a depolarizing pulse to 40 mV to inactivate the HERG channels, followed by different repolarizing pulses to test potentials between −120 and 20 mV in 10 mV steps. The prepulse potential at 40 mV was positive enough to induce full conductance of the HERG channels but also inactivated many of the channels. The rate of recovery from inactivation was obtained by fitting a single exponential to the initial increase in tail-current amplitude, whereas the time constant of deactivation was ascertained by fitting a single exponential to the decay of the tail current. However, 5 *μ*M GA did not change the deactivation rate significantly (*P* > 0.05, *n* = 5) (Figure 6(d)).

## 4. Discussion

Cardiac K^+^ channels play a pivotal role in maintaining normal cardiac electrical activity. They regulate the resting membrane potential and excitability, participate in repolarization, and determine the shape and duration of cardiac action potential. A malfunction of the K^+^ channels due to either gene mutations or drug blockade alters not only cardiomyocyte excitability but also the electrical balance of depolarization and repolarization, which causes a long or short QT interval in the electrocardiogram (ECG) and underlies different types of cardiac arrhythmia [21, 22]. Therefore, cardiac K^+^ channels are important targets for antiarrhythmic drugs. 

In the present study, we have firstly provided the evidence that the antiarrhythmic ionic mechanism of GA is related to the inhibition of potassium currents (*I*_Kr_, *I*_Ks_) in guinea pig ventricular myocytes. The cardiac ion channel gene products that are targets for GA are unknown. However, previous studies have demonstrated that the peak and late *I*_Na_, studied in the Xenopus oocytes expressing either human Na_V_1.5 or mutant Na_V_1.5-ΔKPQ of the *α*-subunit channel, were strongly reduced by GA [19], resulting in a prolongation of the action potential. We therefore tested GA for a possible block of HERG expressed in HEK293 cells. The results confirmed that GA blocks HERG.

One of the main objectives of studying ionic channels is to provide a theoretical basis for the clinical treatment of tachyarrhythmia. Many active ingredients of Chinese medicine could block the HERG channel, decrease *I*_K_, and lead to the acquired long QT syndrome, which makes the channel the therapeutic target for anti-arrhythmia [23]. It is thought that HERG channel inactivation is important in channel blocking by some (but not all) drugs, either by increasing drug-binding affinity or by facilitating the optimal orientation of the S6 aromatic residues to which drugs bind. In this study, the extent of channel inactivation was significantly altered (Figure 5(c)), and the time course of inactivation seemed to be accelerated (Figure 5(b)). Furthermore, the time constants for the onset of inactivation were significantly smaller following perfusion with GA (Figure 6(c)). These effects of GA on channel kinetics were consistent with affinity for the inactivated state, suggesting that GA blocks the HERG channel by affecting its inactivation but not its activation. Our results demonstrated that GA has inhibitory effects on *I*_Kr_, *I*_Ks_ in guinea pig ventricular myocytes, and the HERG potassium channel, and the inhibition was in a concentration-dependent manner. The increase in action potential duration induced by GA is mainly due to its blocking effects on *I*_Kr_, *I*_Ks_, and HERG and are its major mechanisms of antiarrhythmic action. 

*I*_Kr_ and *I*_Ks_ are important in cardiac repolarization [24]. *I*_Kr_ channels open rapidly upon depolarization of the action potential but are quickly inactivate. The channel inactivation is released following repolarization with a slow deactivation [25]. Owing to this inward rectification property, *I*_Kr_ contributes a little during the plateau of the cardiac action potential and progressively increases at phase 3 repolarization of the action potential [26]. Therefore, *I*_Kr_ is pivotal in cardiac repolarization, especially in the later phases of the action potential, due to its unique kinetics. *I*_Ks_ activates slowly with almost no inactivation after activation [25, 27] and contributes to the phase 2 slow repolarization of cardiac action potential. *I*_Ks_ has been demonstrated in cardiac tissues/myocytes from various species including human [24, 26, 27]. The physiological contribution of *I*_Ks_ to the human ventricular action potential is limited; however, during tonic sympathetic stimulation or when the cardiac repolarization reserve is attenuated, *I*_Ks_ becomes important in limiting APD prolongation owing to its slow deactivation [26]. *I*_Ks_ is expressed heterogeneously in different regions of the heart. In the canine ventricle, *I*_Ks_ density is greater in epicardial and endocardial cells than in the M cells [28, 29]. This lower *I*_Ks_ density in the M cells is considered to be related to the steeper APD rate relationship and their greater tendency to display longer APD and to develop EADs at slow heart rates or in response to QT-prolonging drugs [29].

Any abnormality in channel density or function (up- or downregulation) may result in changes of currents and APD, even inducing arrhythmias. The inhibition of *I*_Kr_, *I*_Ks_, and HERG by GA would induce a prolongation of APD, which could contribute to its antiarrhythmic actions because prolongation of APD could prevent or terminate the reentrant excitation and prolong the refractory period. Class III antiarrhythmic agents, blockers of *I*_Kr_ such as d-sotalol, exert a proarrhythmic effect with reverse frequency-dependent manner, which is largely attenuated at fast rates and enhanced at lower stimulation frequencies. This reverse frequency-dependent effect [30] could lead to an increase in the dispersion of repolarization and favor the occurrence of cardiac arrhythmias. Jurkiewicz and Sanguinetti [31] proposed that the reverse frequency-dependent effect on APD of typical class III agents is a consequence of selective blockade of *I*_Kr_. *I*_Kr_ blockers prolong atrial and ventricular APD and the QT interval and can cause TdP, which can degenerate into ventricular fibrillation and sudden cardiac death [2]. Proarrhythmia induced by *I*_Kr_ blockers is related to [32–35] (a) excessive prolongation of APD near plateau voltages, which favor the development of early after-depolarizations; (b) a more marked prolongation of the APD in M cells than in subepicardial or subendocardial ventricular muscle, possibly because of the relative scarcity of *I*_Ks_ in M cells [29]. Thus, triggered focal activity and ventricular reentry associated with increased inhomogeneity of repolarization across the ventricular wall would lead to the development of TdP [3, 4]. It has been suggested that *I*_Ks_ accumulation at increased frequencies decreases the relative importance of *I*_Kr_, reducing the impact of *I*_Kr_ blockade on APD prolongation. The authors suggested that the compounds which inhibit *I*_Ks_ might be devoid of reverse use-dependence. Actually, the agent that blocks both components of *I*_K_ might have a more consistent effect on action potentials at different frequencies and a better safety profile than a specific *I*_Kr_ blocker [36].

Control of cardiac electrical activity is well organized by an array of ion channels activated with a delicate balance between inward and outward ion currents [37]. Upon receiving an incoming impulse, cardiac cells are excited with rapid membrane depolarization followed by a relatively slow repolarization. Repolarization disorders, either excessive slowing or acceleration of the rate, can cause electrical perturbations resulting in cardiac arrhythmias, while excessive blockade of the HERG channel might increase the risk of arrhythmogenic activities. A good antiarrhythmic drug should affect multiple channels and keep invalids free from further episodes of arrhythmia [23]. Taking this into consideration, the drugs currently available in clinics are not satisfactory. Previous studies have revealed that GA can block peak and late *I*_Na_ [19, 20]. In our task group, the results suggested that GA not only blocks *I*_Kr_ (HERG) and *I*_Ks_ but also inhibits *I*_Ca-L_. The effect of GA on multiple channels might make it a promising antiarrhythmic that can lead the cardiac cell to restore normal sinus rhythm and prevent further arrhythmia. Further basic and clinical studies will be needed to explore whether GA has proarrhythmic actions. 

## 5. Conclusion 

In conclusion, our study demonstrated that GA has an inhibitory effect on *I*_K_ (*I*_Kr_, *I*_Ks_) and the HERG potassium channel expressed in HEK293 cells. The results indicate that the antiarrhythmic activity and prolonged action potential of GA could be due to the blocking of *I*_K_ (*I*_Kr_, *I*_Ks_) and the HERG channel. It is thought that a good antiarrhythmic drug should affect multiple channels and be able to restore normal sinus rhythm and keep patients free from further episodes of arrhythmia. GA not only blocks the HERG channel, *I*_K_ (*I*_Kr_, *I*_Ks_), but also inhibits *I*_Na_, so the results reveal that it has significant potential for development as a novel antiarrhythmic agent, particularly targeting the genesis of arrhythmias. Therefore, our findings might help to elucidate the traditional use of licorice in the treatment of cardiovascular disorders. Nonetheless, further evaluation of the therapeutic potential of GA is warranted.
